# The Influence of Growth Milk Consumption on Nutritional Status, Illness Incidence, and Cognitive Function of Children Aged 2–5 Years

**DOI:** 10.3390/children12050545

**Published:** 2025-04-24

**Authors:** Dian Novita Chandra, Kinandra R. K. Rambey, Ifana Aprilliyani, Luthfi Saiful Arif, Rini Sekartini

**Affiliations:** 1 Department of Nutrition, Faculty of Medicine, Universitas Indonesia, Jakarta 10430, Indonesia; dian.chandra@ui.ac.id; 2 Faculty of Medicine, Universitas Indonesia, Jakarta 10430, Indonesia; kinandra.rafa@ui.ac.id (K.R.K.R.); ifana.aprilliyani@ui.ac.id (I.A.); 3 Medical Education Center, Indonesian Medical Education and Research Institute, Faculty of Medicine, Universitas Indonesia, Jakarta 10430, Indonesia; 4 Department of Child Health, Faculty of Medicine, Universitas Indonesia, Jakarta 10430, Indonesia; rini.sekartini@ui.ac.id

**Keywords:** growth, milk, nutritional status, child nutrition, height, cognitive function

## Abstract

**Background**: Adequate nutrition in early childhood is crucial for growth and development. Growth milk, a fortified milk product, has been suggested to address nutritional gaps, but its effectiveness remains uncertain. **Methods**: This clustered randomized controlled trial aimed to assess the effects of growth milk on the nutritional status, immune resilience, appetite, and cognitive function of children aged 2–5 years in Kampung Melayu, East Jakarta. The intervention lasted three months, with 49 participants from two clusters being randomly assigned to either the intervention group or the control group. Nutritional status was assessed monthly. Illness incidence, appetite, food fussiness, and cognitive function were assessed at baseline and endline. **Results**: Children in both groups showed significant improvements in weight, height, and head circumference growth. Significant height differences between the groups was found, although considered minimal clinically. The height-for-age Z-scores significantly increased from −1.65 to −1.58 only in the intervention group, suggesting the positive effects of growth milk supplementation on children’s growth. No significant differences were observed between the groups pertaining to illness incidence, appetite, food fussiness, and cognitive function. Both groups exhibited similar levels of appetite and reported similar cognitive development outcomes. **Conclusions**: Growth milk supplementation resulted in improved growth parameters in children; however, it showed the same trend in the growth milk and the control group. No changes in illness incidence and cognitive development were observed in both groups. Longer studies and the inclusion of malnourished children may provide a better understanding of the broader benefits of growth milk supplementation.

## 1. Introduction

Child malnutrition has been a global burden for many decades. In 2022, the World Health Organization reported that, among children under five years of age, 148.1 million exhibited stunting, while 45 million exhibited wasting [1]. Studies have shown that malnourished children are more likely to experience higher rates of mortality and morbidity, as well as suboptimal cognitive and motor development [2]. Although declines in a child’s growth can be recognized and prevented through the regular assessment of physical indicators, such as height-for-age or weight-for-age Z-scores, malnutrition remains a significant threat [3].

Indonesia has the fourth-largest child population globally, with 80 million children, 10.91% of whom are aged 0–6 years [4]. This age group represents a crucial phase of early childhood and is often referred to as the “golden period”. Although Indonesia has a large number of children under the age of five, a study by Andriani et al. showed that Indonesian children continue to endure malnutrition. Data from the Indonesia Health Research (Riset Kesehatan Indonesia) in 2023 revealed that the prevalence of malnutrition among children under the age of five remains high, with 21.5% exhibiting stunting and 8.5% exhibiting wasting [5]. These conditions highlight the importance of ensuring adequate nutrition during this critical stage to support a strong immune system and cognitive development, which are fundamental to a child’s future well-being [6].

One significant challenge in overcoming malnutrition is picky eating behavior, affecting 25–53% of children globally [7]. Picky eaters are at a higher risk of undernutrition, as they tend to have a lower body weight and a shorter stature than non-picky eaters [7,8]. Deficiencies in essential macronutrients and micronutrients can result in malnutrition, weakening the immune system, reducing appetite, and impairing cognitive development [6,7,8]. In Indonesia, a local study reported that 52.4% of preschool children were picky eaters. Among these children, 44.5% experienced mild-to-moderate malnutrition, underscoring the potential health risks associated with picky eating habits [9]. These negative consequences may persist even in adulthood, emphasizing the importance of early nutritional interventions [8].

To address nutritional deficiencies, dietary supplementation, including milk consumption, has been widely recommended. A study by Herber et al. (2020) reported that milk consumption is associated with a reduced probability of being underweight by 1.4 percentage points and a reduced probability of being stunted by 1.9 percentage points, suggesting a potential protective role in child growth outcomes [10]. Moreover, fortified milk, a formula milk product containing essential nutrients such as proteins, vitamins, and minerals with fish oil and probiotic fortification, could aid in fulfilling children’s nutritional needs [11]. Although systematic reviews and meta-analyses suggest that fortified milk can help bridge nutrient gaps, its effects on weight gain and height improvement remain minimal [12]. Moreover, research on formula milk has raised concerns regarding potential health risks, including gastrointestinal infections and respiratory diseases, with some claims about cognitive benefits still being debated [13].

Given the conflicting research findings, further investigation is necessary to assess the role of growth milk in supporting children’s health. This study aims to evaluate the effects of growth milk consumption on nutritional status, immune resilience, appetite, and cognitive function of children aged 2–5 years, addressing existing knowledge gaps and contributing to evidence-based nutritional recommendations.

## 2. Materials and Methods

### 2.1. Study Design and Sample

This study was an experimental study employing a clustered randomized controlled trial design. The study was conducted at Kampung Melayu Sub-district Community Health Center (Puskesmas), East Jakarta, which covered 12 community groups (*rukun warga*/RW). The randomization was performed using simple random sampling to choose 2 RWs as the intervention and control groups. This study only included two RWs, which were located far apart from each other to prevent the risk of participants in different groups influencing each other. The intervention lasted three months, from September 2024 to November 2024. The target population consisted of children aged 2–5 years residing in the selected RWs in Kampung Melayu. Enrollment was conducted at the integrated service post by health cadres.

**Figure 1:**
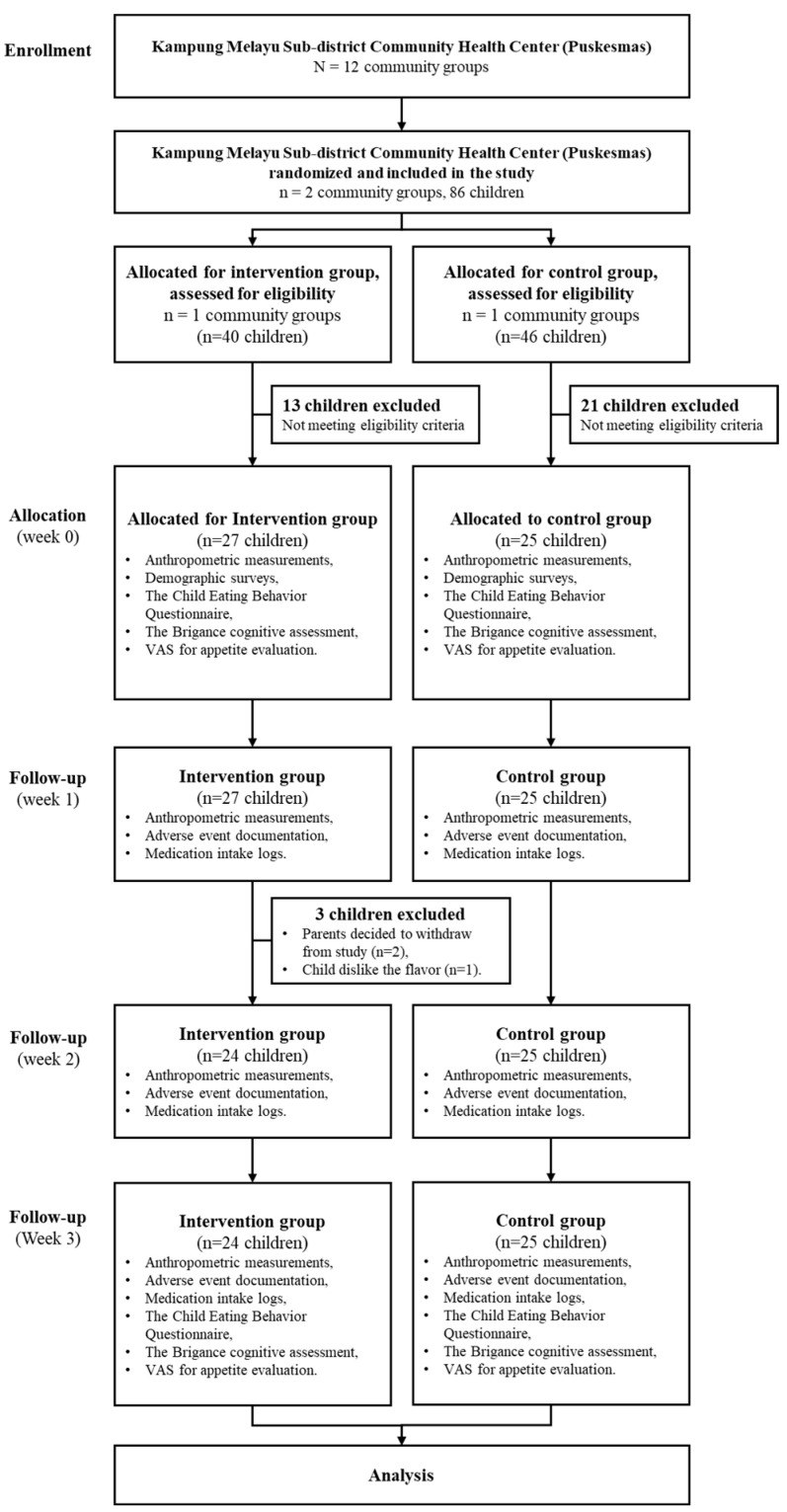
Consort diagram for 3-week milk intervention.

Using a priori power analysis, the sample size was determined to be 25 subjects per group, based on the independent two-population mean formula, with a significance level of 0.05 and a power of 95%, guided by prior research findings. A total of 52 children were recruited, with 25 participants in the control group and 27 in the intervention group. Two subjects from the intervention group withdrew from the study due to parental withdrawal of consent, and one child refused to continue because they disliked the taste of the milk. For this reason, a post hoc power analysis was conducted based on an expected mean difference in height-for-age Z-scores (HAZ) of 1.47 ± 0.83 in the intervention group and 0.93 ± 0.40 in the control group, yielding an estimated effect size (Cohen’s d) of 0.84. This sample size provided an actual statistical power of approximately 84.3%. Figure 1 illustrates the sample recruitment process for the study.

### 2.2. Inclusion and Exclusion Criteria

Participants eligible for inclusion in this study were children aged 2–5 years who were in good health at the time of enrollment. They were required to have a weight-for-height measurement between 0 and −2 SD on the WHO growth curve and should not have had a habit of consuming growth milk regularly, defined as less than four times per week. Only children whose parents provided informed consent and agreed to participate in the study were included. The drop out criteria consisted of participants who were lost to follow-up during the study period or those who failed to adhere to the prescribed milk consumption protocol. Children who experienced significant health issues unrelated to the study intervention that could impact their nutritional status were also excluded from the analysis.

### 2.3. Intervention and Data Collection Procedures

Parents of eligible children were provided with a detailed explanation of the study and were required to sign an informed consent form before participation. Baseline anthropometric assessments, including weight, height, and head circumference, were conducted using standardized equipment. Trained enumerators conducted interviews and administered pre-intervention questionnaires, including demographic surveys, the Child Eating Behavior Questionnaire for food fussiness, and the Brigance cognitive assessment, along with a Visual Analog Scale (VAS) for appetite evaluation. Appetite towards food was also evaluated to distinguish the effects of milk consumption and higher milk intake.

The intervention group received growth milk powder in a sachet (39 g per sachet). Each sachet was diluted with warm water to obtain one serving (approximately 200 mL of milk), and participants were instructed to consume two servings per day (400 mL in total) for three months. Milk sachets were distributed weekly, and community health workers monitored adherence through weekly check-ins. The milk composition was provided in Supplementary Table S1. Parents were required to return empty sachets, and any remaining unused sachets were also collected. The control group received only nutritional education without any milk supplementation. Nutrition education, focused on the importance of a balanced nutritional intake, was provided through a PowerPoint presentation to the parents from control and interventional group before the recruitment started. During this session, parents were advised to maintain their children’s usual dietary patterns without modifications if they were chosen in the study.

### 2.4. Follow-Up and Monitoring

Daily follow-ups were conducted via telephone to ensure compliance with the milk consumption protocol. Weekly compliance forms were recorded, and monthly assessments included adverse event documentation and medication intake logs. Anthropometric measurements were repeated at baseline, one month, two months, and three months post-intervention. Final assessments included the same set of questionnaires and VAS evaluation. All health conditions of the participants were closely monitored throughout the study. Parents or caregivers were interviewed regarding the child’s health status on each visit. Any illnesses or complaints, whether related to the intervention or not, were documented on the adverse event recording form. Any medications or therapies received outside the study intervention were separately recorded. In cases of severe illness requiring hospitalization, researchers reported the event to the ethics committee within 24 h.

### 2.5. Data Analysis Plan

All collected data were securely stored digitally and analyzed using univariate, bivariate, and multivariate statistical methods to assess relationships between independent and dependent variables. Statistical analyses were performed using SPSS 22.0 for Windows.

## 3. Results

### 3.1. Study Population

The study involved a total of 49 children aged 2 to 5 years, with 25 participants from the cluster assigned to the control group and 24 participants from the cluster receiving the intervention of growth milk. The demographic characteristics of the participants, including age, gender distribution, and baseline anthropometric measurements, such as weight and height, were comparable between the two groups. Statistical analyses confirmed that there were no significant differences in these baseline characteristics, ensuring that any observed effect could be attributed to the intervention rather than pre-existing disparities (summarized in Table 1).

### 3.2. Growth Outcomes

There were no significant differences in height, weight, Z-score, and head circumference between the intervention and the control group, as shown in Table 2 and Table 3. However, over the three-month intervention period, significant improvements were observed in several growth parameters of both groups. The average weight of children in the intervention group increased from 12.91 kg at the baseline to 13.32 kg at the end of the study, reflecting a mean weight gain of 0.41 kg (*p* < 0.001). Similarly, a weight increase was also observed in the control group with an average weight of 12.48 kg at the baseline to 12.98 kg at the end of the study, reflecting a mean weight gain of 0.50 kg (*p* < 0.001). This weight gain is indicative of the improved nutritional status and growth. However, if the change in body weight was compared between the intervention and the control group, no significant difference was found (*p* < 0.573). Similarly, height measurements in each group showed a significant increase, with the average height in the intervention group increasing from 94.61 cm at the baseline to 96.54 cm at the end of the study, representing a mean increase of 1.93 cm (*p* < 0.001), while the average height in the control group increased from 92 cm at the baseline to 94 cm at month three, although the comparison of two groups did not show a significant difference.

### 3.3. Z-Score Analysis

Height-for-age and body mass index (BMI)-for-age Z-scores were also analyzed to more comprehensively assess the nutritional status of the children. The intervention group exhibited significant improvements in their height-for-age Z-scores, which increased from −1.65 to −1.58 over the study period, indicating a positive shift towards normal growth patterns, but no statistical difference was observed when compared with the control group (summarized in Table 2). Furthermore, after performing an analysis exclusively within each group, we found a statistically significant increase in the height-for-age parameter when comparing the baseline to first month (0.11 ± 0.16; *p* = 0.004), baseline to second month (0.12 ± 0.20; *p* = 0.007), and baseline to third month (0.15 ± 0.30; *p* = 0.023) only among the intervention group as demonstrated in Table 3. Meanwhile, the BMI-for-age Z-scores showed a similar trend for the intervention group, demonstrating a significant increase, thereby suggesting that the growth milk not only supported weight gain but also contributed to healthier body composition. Furthermore, head circumference measurements indicated a notable increase, with the average head circumference increasing from 47.73 cm to 48.54 cm, reflecting significant changes in brain growth and development in both groups (summarized in Table 2 and Table 3).

### 3.4. Health and Cognitive Outcomes

Despite the positive outcomes for growth parameters, this study did not find significant differences in health-related outcomes between the two groups. The incidence of illness, as recorded during the study, was similar in both the control and intervention groups, with an average of 1.44 episodes of illness reported in the control group compared to 1.67 in the intervention group (*p* = 0.654). There was no significant difference observed for specific illness incidence as summarized in Table 4. Additionally, appetite scores measured using the Visual Analog Scale (VAS) showed no significant changes, with both groups reporting similar levels of appetite, or liking looking at food, throughout the study. Food fussiness, assessed using the Child Eating Behavior Questionnaire (CEBQ), also did not reveal significant differences, indicating that the intervention did not alter children’s eating behaviors (summarized in Table 4). Cognitive function, evaluated using the Brigance screening tool, showed no significant differences between the intervention and control groups after the study, suggesting that, while growth parameters improved, cognitive development remained unaffected by the growth milk intervention. Language scores declined slightly in both groups, but the difference was not significant (*p* = 0.925).

## 4. Discussion

This study investigated the effect of growth milk compared to no additional milk on the nutritional status, immune resilience, appetite, and cognitive function of children aged 2–5 years. We found that providing 400 mL of growth milk per day for three months is associated with significant improvements in anthropometric measures. Subject control also showed improvements in anthropometric measures, but the same as with the intervention group. Although both groups exhibit a statistically significant increase in their weight and height based on the normal growth pattern, the notable increase in BMI-for-age Z-scores and height-for-age Z-scores is only statistically significant for the intervention group. The findings of this study are consistent with those of previous studies on the effect of milk supplementation on the anthropometric status of children [12,14,15]. According to the Center of Disease Control and Prevention, the average growth in height of children aged 2–5 years is approximately 0.43–0.64 cm per month, while the average weight gain is about 0.19–0.23 kg per month [16,17]. Children in the intervention group managed to show the expected average increase in height and weight and even exceeded it. Although in the comparison between control and intervention group significance height difference was found in the second month, this difference was considered minimal clinically and still can be assumed to be in line with the child growth trajectory based on WHO child growth standards [3]. The role of growth milk in height gain can be attributed to its association with a higher concentration of blood calcium, which suggests that nutrients in milk are essential to improve bone mineralization and achieve better bone density [18]. A previous study analyzing the dietary intake of Indonesian children aged 1–5 years observed that over 50% of the study population had inadequate intakes of iron, calcium, zinc, and vitamins A, D, and B [19].

Data from SEANUTS II Indonesia indicate a high prevalence of micronutrient deficiencies among children under the age of five years, with 22.8% of these children exhibiting anemia, 20.3% iron deficiency, 11.9% zinc deficiency, 1.9% vitamin A deficiency, and 27.1% vitamin D insufficiency [20]. Given this substantial burden, several studies have shown that growth milk consumption can help improve micronutrient intake. A study in China found that growth milk consumption significantly enhanced nutrient intake, while dietary modeling in the Philippines demonstrated that adding growth milk to the diet increased vitamin and mineral intake [21,22]. Additionally, a randomized controlled trial in New Zealand reported lower iron and vitamin D deficiency rates among growth milk consumers [23]. These findings suggest that, although this study did not find a significant difference in weight gain between the two groups, growth milk has been shown to improve micronutrient status. This highlights the need for further research to explore its role in addressing micronutrient deficiencies in young children.

Growth milk used in this study is rich in nutrients required during bone formation, which enhance height gain. Studies conducted in developing countries usually revealed slightly higher growth outcomes after the supplementation of growth milk, likely because children in such countries generally experienced compromised development prior to intervention [9]. Although the gain in weight corresponds to the growth in height, evidence found in this study suggested that growth milk does not promote dramatic weight gain leading to obesity, as reflected by relatively stable weight-for-age Z-scores throughout the three months. However, the available literature may show incongruence in results. A similar study performed in India found that the group receiving fortified milk exhibited more height gain when compared to the control group [24], and a similar result was reported by a study performed in The Philippines, which noted that normo-weight subjects receiving fortified milk were at risk of becoming overweight within 12 weeks of the intervention [8]. However, previous systematic reviews found no significant impacts of fortified milk on height gain [12,25]. This may be due to differences in nutrients present in the fortified milk used in each intervention. Additionally, when compared to WHO’s anthropometric indices, a study in Nigeria found that subjects consuming 600 mL fortified milk for 6 months showed HAZ improvements; however, the results were not statistically significant among groups [26]. Differences in demography and protocols may also account for discrepancies among studies. Moreover, although powdered milk contains a perfectly balanced ratio of proteins, fats, carbohydrates, antibodies, and enzymes that support a baby’s growth and immune system, it has a higher proportion of casein protein. Powdered milk often contains added DHA, ARA, and probiotics, but they are not naturally absorbed in a high proportion by the body [27,28].

While children in the intervention group showed a notable increase in head circumference of 0.81 ± 0.75 cm, we found no difference in cognitive parameters of the children before and after the study. Additionally, growth milk supplementation did not bring about changes in eating behavior. It has been suggested that vitamin D present in growth milk, responsible for processes such as immunomodulation and nerve conduction, is pivotal in the development of the brain, but these findings were inconclusive and still need to be investigated further [29]. While PUFA and DHA concentrations in growth milk may be positively associated with better cognitive outcomes, such as higher concentration and memory, their relationship with overall cognitive performance remains to be investigated [9,21]. Notably, aside from nutrition, other factors, such as physical activity, sleep quality, and socioeconomic status, contributed to the brain’s cognitive function, i.e., children’s ability to learn [30]. Growth milk is rich in immune-supporting micronutrients, and the insignificant effect of growth milk on the incidence of illness demonstrated in our study may hint at the normal nutrition status of children prior to supplementation and the older age of the children, as the participants of previous studies that reported the beneficial effects of growth milk in lowering morbidity comprised malnourished children or children younger than two years [31,32]. A study investigating the effect of changes in dietary patterns in undernourished children revealed an increase in the number of activated B cells and pro-inflammatory cytokines, but no changes in other cells involved in adaptive immunity [33].

Despite these differences, evidence from other studies supports the role of growth milk in reducing illness. A retrospective cohort clinical study conducted in Indonesia demonstrated that the daily consumption of at least 500 mL of growth milk for a minimum of six months could result in a 58% reduction in the incidence of acute respiratory infections (ARIs), suggesting that the regular consumption of growth milk may support immune system development and protection against infections [34]. Similarly, a study conducted in India found that the administration of growth milk, administered as three sachets per day for one year, significantly reduced morbidity in preschool children, including a decrease in the incidence of diarrhea (18%), pneumonia (26%), high fever (7%), and the number of sick days due to severe illness (15%) [32]. Both studies had longer intervention durations and a higher milk intake, which may explain why they observed significant reductions in morbidity, whereas our study did not demonstrate similar findings.

The results of this study can provide valuable insights for parents and healthcare professionals regarding the role of milk in child growth. A 3-month milk intervention showed no significant effects on weight and body mass index in the intervention group compared to the control group. Therefore, health professionals should educate parents not to expect rapid improvements from milk consumption alone. Other factors that influence a child’s growth, such as individual characteristics, should also be considered. Moreover, it is important to promote the understanding that milk supplementation must be accompanied by adequate overall nutrition to help children reach their full growth potential.

This study has several limitations. This study had a wide age range with no additional analysis conducted in sub-group based on age. Future studies should include more participants, with robust calculations to ensure the statistical power for each age sub-group. The relatively short duration (3 months) may not be sufficient to observe significant growth differences, requiring a longer observation period. Environmental factors also play a crucial role. A flood disaster in the third month affecting the intervention group may have increased diarrhea cases, which could impede growth. Additionally, factors such as physical activity, sedentary lifestyle, and sleep patterns were not assessed but could influence growth. Irregular sleep can affect growth hormone production, while a lack of physical activity and sun exposure and a sedentary lifestyle may impair muscle and bone development. A previous study suggested making comparisons between seasons due to the higher risk of respiratory infections and a decline in immunity during late autumn and winter. Although Indonesia is located on the equator and only has two seasons, it is still interesting to further explore differences in children’s immunity between the rainy and dry seasons in future studies. These external factors should be considered when evaluating the effectiveness of milk supplementation on child growth.

One important factor that was not considered in the sample selection is breastfeeding history, including whether breastfeeding occurred, the type of breastfeeding, and its duration. This could be a significant variable influencing the study’s results, particularly in areas related to child development, health outcomes, and cognitive functions. Breastfeeding provides essential nutrients and immunological benefits that may affect physical growth, immune system strength, and overall health. Additionally, some studies suggest a link between breastfeeding and improved cognitive development, which could be a confounding factor in research examining learning and intelligence [35].

Not accounting for breastfeeding history may introduce variability into the findings, potentially affecting the reliability of drawn conclusions. Differences in breastfeeding exposure among participants could create biases that were not controlled for, influencing observed outcomes. Thus, the omission of breastfeeding history as a criterion represents a limitation of this study. Future research should consider incorporating breastfeeding variables to better understand their potential effects on the investigated outcomes [35].

## 5. Conclusions

This study found that growth milk supplementation resulted in improved growth parameters, with the same trend observed in both the growth milk and the control group. Although statistically significant, difference in height growth between both group was considered minimal based on child growth standard. Furthermore, no difference in illness incidence and cognitive development was observed in both groups. Future research should consider longer studies and the inclusion of malnourished children to provide a better understanding of the benefits of growth milk supplementation. This study provides valuable insights that can be applied in practical settings, particularly in Indonesia. By addressing milk consumption, this study offers actionable recommendations for mothers. These insights can help improve the nutritional status of children, making the study’s contribution more tangible and applicable.

## Figures and Tables

**Table 1:** Children’s demographic data.

	Control (n = 25)	Intervention (n = 24)	*p*-Value
Gender, n (%)			0.482
Male	10 (45.45)	12 (54.55)	
Female	15 (55.56)	12 (44.44)	
Age (years, mean ± SD)	3.56 ± 1.00	3.86 ± 0.85	0.354 ^a^
Age (years, median (min–max))	3.58 (2.11–5.67)	3.90 (2.54–5.53)	0.354 ^a^
Birth weight (gram, mean ± SD)	3028 ± 492	2989 ± 506	0.637 ^b^
Prematurity status			-
Normal	25 (51.02)	24 (48.98)	
Premature	0 (0.00)	0 (0.00)	
Child status			-
Biological child	25 (51.02)	24 (48.98)	
Adopted child	0 (0.00)	0 (0.00)	

Notes: significant at *p* < 0.05; ^a^ independent *t*-test; ^b^ Mann–Whitney test.

**Table 2:** Comparison of children’s weight, height, Z-score, and head circumference over 3 months.

	Intervention (n = 24)	Control (n = 25)	*p*-Value
Weight (kg)			
At baseline (month 0)	12.91 ± 1.71	12.48 ± 2.23	0.456 ^a^
Month 1	13.02 ± 1.59	12.60 ± 2.24	0.455 ^a^
Month 2	13.04 ± 1.58	12.81 ± 2.41	0.702 ^a^
Month 3	13.32 ± 1.55	12.98 ± 2.51	0.573 ^a^
Height (cm)			
At baseline (month 0)	94.61 ± 7.16	92.61 ± 8.09	0.364 ^a^
Month 1	95.54 ± 6.87	93.22 ± 8.07	0.286 ^a^
Month 2	96.08 ± 6.88	93.53 ± 7.95	0.238 ^a^
Month 3	96.54 ± 6.74	94.24 ± 8.11	0.288 ^a^
Weight-for-age			
Z-Score for month 0	−1.65 ± 0.58	−1.69 ± 0.88	0.860 ^a^
Z-Score for month 1	−1.64 ± 0.53	−1.69 ± 0.88	0.809 ^a^
Z-Score for month 2	−1.71 ± 0.50	−1.66 ± 0.95	0.823 ^a^
Z-Score for month 3	−1.58 ± 0.50	−1.64 ± 0.95	0.787 ^a^
Height-for-age			
Z-Score for month 0	−1.73 ± 0.80	−1.78 ± 0.98	0.862 ^a^
Z-Score for month 1	−1.62 ± 0.75	−1.76 ± 0.95	0.579 ^a^
Z-Score for month 2	−1.61 ± 0.76	−1.81 ± 0.93	0.427 ^a^
Z-Score for month 3	−1.58 ± 0.73	−1.76 ± 0.98	0.482 ^a^
Body mass index-for-age			
Z-Score for month 0	−0.78 ± 0.46	−0.78 ± 0.50	0.998 ^a^
Z-Score for month 1	−0.89 ± 0.52	−0.81 ± 0.51	0.596 ^a^
Z-Score for month 2	−1.00 ± 0.53	−0.71 ± 0.61	0.073 ^b^
Z-Score for month 3	−0.84 ± 0.61	−0.74 ± 0.61	0.536 ^a^
Head circumference (cm)			
At baseline (month 0)	47.73 ± 1.50	47.41 ± 1.71	0.489 ^a^
Month 1	48.21 ± 1.41	47.90 ± 1.49	0.465 ^a^
Month 2	48.48 ± 1.46	48.11 ± 1.44	0.382 ^a^
Month 3	48.54 ± 1.45	48.34 ± 1.36	0.621 ^a^

Notes: significant at *p* < 0.05; ^a^ independent *t*-test; ^b^ Mann–Whitney test.

**Table 3:** Comparison of children’s weight and height, Z-score, and head circumference among children over 3 months.

	Intervention (n = 24)	*p*-Value (Same Group)	Control (n = 25)	*p*-Value (Same Group)	*p*-Value (Between Groups)
Weight (kg)					
Month 0 and month 1	0.11 ± 0.34	0.121 ^a^	0.12 ± 0.21	0.010 ^a^*	0.926 ^c^
Month 0 and month 2	0.13 ± 0.39	0.120 ^a^	0.33 ± 0.44	0.001 ^a^*	0.095 ^c^
Month 0 and month 3	0.41 ± 0.44	<0.001 ^a^*	0.50 ± 0.58	<0.001 ^a^*	0.562 ^c^
Height (cm)					
Month 0 and month 1	0.93 ± 0.68	<0.001 ^a^*	0.61 ± 0.38	<0.001 ^a^*	0.123 ^d^
Month 0 and month 2	1.47 ± 0.83	<0.001 ^a^*	0.93 ± 0.40	<0.001 ^a^*	0.013 ^d^*
Month 0 and month 3	1.93 ± 0.93	<0.001 ^a^*	1.64 ± 0.54	<0.001 ^a^*	0.190 ^c^
Weight-for-age					
Z-Score for month 0 and month 1	0.01 ± 0.23	0.887 ^a^	−0.06 ± 0.14	0.829 ^a^	0.813 ^c^
Z-Score for month 0 and month 2	0.06 ± 0.26	0.272 ^a^	0.03 ± 0.26	0.614 ^a^	0.069 ^d^
Z-Score for month 0 and month 3	0.06 ± 0.32	0.353 ^a^	0.04 ± 0.32	0.517 ^a^	0.749 ^d^
Height-for-age					
Z-Score for month 0 and month 1	0.11 ± 0.16	0.004 ^a^*	0.02 ± 0.10	0.373 ^a^	0.026 ^c^*
Z-Score for month 0 and month 2	0.12 ± 0.20	0.007 ^a^*	−0.03 ± 0.11	0.194 ^a^	0.002 ^c^*
Z-Score for month 0 and month 3	0.15 ± 0.30	0.023 ^b^*	0.02 ± 0.14	0.503 ^a^	0.112 ^d^
Body mass index-for-age					
Z-Score for month 0 and month 1	0.10 ± 0.37	0.086 ^b^	0.03 ± 0.23	0.528 ^a^	0.374 ^c^
Z-Score for month 0 and month 2	0.22 ± 0.37	0.008 ^a^*	0.07 ± 0.40	0.415 ^a^	0.009 ^d^*
Z-Score for month 0 and month 3	0.06 ± 0.43	0.474 ^a^	0.04 ± 0.45	0.626 ^a^	0.374 ^c^
Head circumference (cm)					
Month 0 and month 1	0.48 ± 0.65	0.001 ^a^*	0.49 ± 0.59	<0.001 ^a^*	0.801 ^d^
Month 0 and month 2	0.75 ± 0.76	<0.001 ^a^*	0.70 ± 0.73	<0.001 ^a^*	0.802 ^d^
Month 0 and month 3	0.81 ± 0.75	<0.001 ^a^*	0.93 ± 0.84	<0.001 ^a^*	0.582 ^d^

Notes: * significant at *p* < 0.05; ^a^ paired *t*-test; ^b^ Wilcoxon test; ^c^ independent *t*-test; ^d^ Mann–Whitney test.

**Table 4:** Comparison of incidence of illness in children, VAS scores for liked looking at food, CEBQ scores for food fussiness, and Brigance scores between control and intervention groups over 3 months.

	Control (n = 25)	Intervention (n = 24)	*p*-Value
Illness incidence	1.44 ± 1.53	1.67 ± 1.66	0.654
URTI	1.08 ± 1.32	1.21 ± 1.18	0.577
Mumps	0.04 ± 0.20	0.04 ± 0.20	0.977
Dermatitis	0.00 ± 0.00	0.08 ± 0.28	0.145
Minor trauma	0.00 ± 0.00	0.04 ± 0.20	0.307
Gastritis	0.00 ± 0.00	0.13 ± 0.61	0.307
Conjunctivitis	0.04 ± 0.20	0.04 ± 0.20	0.977
Diarrhea	0.12 ± 0.44	0.08 ± 0.28	1.000
Toothache	0.08 ± 0.28	0.04 ± 0.20	0.580
Stomatitis	0.08 ± 0.28	0.00 ± 0.00	0.161
VAS scores			
VAS liked looking at food for month 0	9.00 ± 1.66	9.17 ± 1.49	0.685
VAS liked looking at food for month 3	8.84 ± 1.28	9.21 ± 0.98	0.344
Difference in VAS liked looking at food between month 0 and month 3	0.16 ± 1.37	0.04 ± 1.12	0.657
CEBQ scores			
CEBQ score for month 0	2.49 ± 0.65	2.10 ± 0.56	0.052 ^b^
CEBQ score for month 3	2.37 ± 0.51	2.10 ± 0.88	0.199 ^a^
Difference in CEBQ score between month 0 and month 3	−0.12 ± 0.74	0.00 ± 0.74	0.573 ^a^
Brigance scores			
At baseline (month 0)			
Total score	50.41 ± 25.71	54.84 ± 17.14	0.543
Cognitive score	15.79 ± 11.82	17.61 ± 10.52	0.630
Language score	20.82 ± 13.52	23.16 ± 10.71	0.568
Physical score	13.79 ± 7.51	14.08 ± 6.48	0.904
Month 3			
Total score	55.00 ± 26.84	56.53 ± 19.45	0.845
Cognitive score	20.71 ± 12.53	19.45 ± 11.99	0.760
Language score	19.59 ± 14.84	22.26 ± 11.02	0.541
Physical score	14.71 ± 6.76	14.82 ± 7.40	0.963
Score difference			
Total score	4.59 ± 15.68	1.68 ± 21.99	0.655
Cognitive score	4.91 ± 7.42	1.84 ± 7.94	0.241
Language score	−1.24 ± 10.55	−0.89 ± 10.84	0.925
Physical score	0.91 ± 7.22	0.74 ± 9.19	0.950

Notes: significant at *p* < 0.05; ^a^ independent *t*-test; ^b^ Mann–Whitney test.

## Data Availability

The data supporting this study are not publicly available as they are owned by the sponsor.

## Supplementary Materials

The following supporting information can be downloaded at: https://www.mdpi.com/article/10.3390/children12050545/s1, Table S1: Growth Milk Nutrition Information.

## Abbreviations

The following abbreviations are used in this manuscript:

VAS	Visual Analog Scale
CEBQ	Child Eating Behavior Questionnaire
BMI	body mass index
RW	*Rukun Warga* (Neighborhood Administrative Unit)
