# Supplementary material for: The Influence of Growth Milk Consumption on Nutritional Status, Illness Incidence, and Cognitive Function of Children Aged 2–5 Years
Source: Children (Basel). 2025 Apr 24;12(5):545. doi: 10.3390/children12050545 (PMC12109987; doi:10.3390/children12050545)
Supplement: Supplementary file 1 [file children-12-00545-s001.zip › children-3565482-supplementary.pdf]

Supplementary Table S1. Growth Milk Nutrition Information

No.	Category	Composition	Amount per serving	%AKG
1.	Total Energy		180 kkal	
2.	Energy from fat		60 kkal	
3.	Fat	Total fat	7 g	10%
		Trans fats	0 g	
		Cholesterol	5 mg	2%
		AA	7,8 mg	
		DHA	26 mg	
		Fosfolipid	70 mg	
		Omega 6	600 mg	
		Omega 3	100 mg	
		Omega 6 (asam linoleat)	585 mg	5%
		Omega 3 (asam alfa-linoleat)	70 mg	5%
		Saturated fat	3 g	15%
4.	Protein	Protein	5 g	9%
		Nucleotides	5,9 mg	
		Taurine	7,8 mg	
5.	Carbohydrates	Total Carbs	24 g	7%
		Dietary fiber	1 g	4%
		FOS	1200 mg	
		Total sugar	5 g	
6.	Vitamin	Vitamin A		25%
		Vitamin C		30%
		Vitamin D		25%
		Vitamin E		20%
		Vitamin K		20%
		Vitamin B1 (tiamin)		20%
		Vitamin B2 (riboflavin)		20%
		Vitamin B3 (Niacin)		20%
		Vitamin B5 (pantothenic acid)		25%
		Vitamin B6 (piridoxin)		20%
		Vitamin B9 (folic acid)		20%
		Vitamin B12 (cobalamin acid)		20%
		Biotin		20%
		Choline		10%
7.	Mineral	Salt (sodium)	150 mg	10%
		Potassium		6%
		Calcium		20%

		Besi		15%
		Phosphorus		20%
		Magnesium		6%
		Zinc		20%
		Copper		8%
		Iodium		10%
		Selenium		15%
		Inositol	10 mg	
8.	Essential amino acids	Total essential amino acids	2150 mg	
		Triptofan	65 mg	
		Histidine	130 mg	
		Threonine	230 mg	
		Valine	290 mg	
		Methionine	130 mg	
		Lysine	330 mg	
		Isoleusin	270 mg	
		Leusin	465 mg	
		Phenylalanine	240 mg	

AKG: The Indonesian Dietary Recommendation
